# Lymph nodes primary staging of colorectal cancer in 18F-FDG PET/MRI: a systematic review and meta-analysis

**DOI:** 10.1186/s40001-023-01124-4

**Published:** 2023-05-04

**Authors:** Qingwei Ren, Yanyan Chen, Xuejun Shao, Lanzhong Guo, Xinxin Xu

**Affiliations:** 1 Department of Gastroenterology, Dongyang Hospital of Traditional Chinese Medicine, Dongyang, China; 2 Department of Medical Oncology, Dongyang Women & Children Hospital, No. 40, Wuning East Road, Dongyang, 322100 China

**Keywords:** PET/MRI, Colorectal, Lymph node metastasis, FDG, Diagnosis, Meta-analysis

## Abstract

**Objective:**

To assess the diagnostic efficacy of 18F-FDG PET/MRI for lymph node (LN) metastasis primary staging in patients with colorectal cancer (CRC).

**Methods:**

This study was conducted and reported in accordance with the PRISMA-DTA statement. Electronic databases (PubMed, Embase, Cochrane Library) were searched for studies on 18F-FDG PET/MRI for diagnosing LN metastasis. The pooled sensitivity (SEN), specificity (SPE), and area under the curve (AUC) were applied to assess the diagnostic performance. Heterogeneity was identified and processed using meta-regression and sensitivity analysis. All data analyses were performed via STATA 15 and Meta-Disc 1.4 software.

**Results:**

There were finally 7 studies included, involving a total of 184 patients. The Spearman rank correlation coefficient was 0.108 (*P* = 0.818), with no threshold-effect observed. The pooled SEN was 0.81 (95%CI 0.66–0.90) and the SPE was 0.89 (95% CI 0.73–0.96). In sub-groups, prospective groups demonstrated to have the highest SEN of 0.92 (95%CI 0.79–1.00). The studies conducted by Catalano et al. and Kang et al. were considered to be potential sources of heterogeneity.

**Conclusion:**

18F-FDG PET/MRI has shown remarkable diagnostic performance in identification of LN metastases in newly diagnosed CRC patients. It would be of great application value for the primary staging of CRC lymph node metastases.

## Introduction

Colorectal cancer (CRC) refers to the most common gastrointestinal malignancy that occurs in the proximal colon, distal colon, or rectum [1], and is the leading cause of cancer-related deaths and the third most common disease worldwide [2]. Surgery and chemotherapy are conventional treatment approaches for CRC. However, the treatment outcomes of CRC remain unsatisfactory due to the high risk of recurrence and metastasis, especially in patients at advanced stage [3, 4].

Imaging plays an increasingly important role in the diagnosis, staging, and prognosis-based treatment stratification of CRC. Lymph node (LN) metastasis is one of the most important prognostic factors for CRC patients [4]. The 5-year survival rate of CRC patients without LN-metastasis exceeds 95%, while that of patients with LN metastasis would decrease by 25%-45% [4]. Early identification of LN metastasis could improve the diagnosis and facilitate the initiation of second-line treatment in CRC patients [3]. Conventional imaging techniques, such as computed tomography (CT) and magnetic resonance imaging (MRI), have been widely applied for CRC assessment [5–7]. F-18 fluorodeoxyglucose (FDG) positron emission tomography/computed tomography (PET/CT) has also shown to be promising for CRC-staging by providing metabolic information of the cancer tissues [8, 9]. Positron emission tomography/magnetic resonance (PET/MRI) is a hybrid imaging method that combines the high soft tissue contrast of MRI with the sensitivity of PET to enable the simultaneous collection of PET and MRI data. Recent studies have demonstrated that 18F-FDG PET/MRI is of great performance in CRC staging [10, 11]. It is capable of regional and full-body scanning in a single session so as to provide more effective and accurate information for diagnosis and staging [12]. More importantly, it is capable of identifying LN metastases and differentiating ambiguous lesions, which could improve the diagnostic accuracy and efficacy [13, 14].

To date, there has been a lack of systematic reviews to assess the diagnostic performance of 18F-FDG PET/MRI for LN-metastatic primary staging in CRC patients. Therefore, we have conducted this systematic review and meta-analysis in the hopes of providing an evidence-based reference for the clinical application of 18F-FDG PET/MRI.

## Materials and methods

This study has been registered in INPLASY. (Number: 2022110141).

### Electronic searches

We searched 3 electronic databases including PubMed, Cochrane Library, and Embase from inception to June, 2022 for relevant studies. Search items mainly included colon, colorectal or rectal, positron emission tomography/magnetic resonance imaging, PET-MRI, and PET-MR. Search strategies for PubMed, Embase and Cochrane Library are shown in Table 1. Reference lists of identified articles were also screened for potential eligible study.

**Table 1:** Search strategy in PubMed, Embase and Cochrane Library

Database	Search strategy
PubMed (112)	("colon*"[All Fields] OR "colorectal"[All Fields] OR ("administration, rectal"[MeSH Terms] OR ("administration"[All Fields] AND "rectal"[All Fields]) OR "rectal administration"[All Fields] OR "rectal"[All Fields])) AND ("PET-MRI"[All Fields] OR "positron emission tomography/magnetic resonance imaging"[All Fields] OR "PET-MR"[All Fields])
Embase (342)	('colon*' OR 'colorectal' OR 'rectal') AND('PET-MRI' OR 'positron emission tomography/magnetic resonance imaging' OR 'PET-MR')
Cochrane Library(50)	(colon* OR colorectal OR rectal) AND(PET-MRI OR "positron emission tomography/magnetic resonance imaging" OR PET-MR)

### Selection of studies

Studies that met the following criteria would be included:

- Assessing the efficacy of 18F-FDG PET/MRI for LN metastasis identification;
- Histopathological results or image follow-up used as golden standard for LN metastasis and TNM staging;
- Complete data available (true positive (TP), true negative (TN), false positive (FP), and false negative (FN));
- Prospective study or retrospective study.

Study reported and published in non-English, literature review, letter to the author, comments, case report or case series, and study with the patients having recognized risk factors would be excluded.

### Quality assessment

Two reviewers independently proceeded the quality assessment of included studies via the Quality Assessment of Diagnostic Accuracy Studies (QUADAS-2), which refers to 4 domains including patient selection, index test, reference standard, and flow and timing. Each domain could be graded as “high”, “low”, or “unclear”. Disagreements were settled via discussion.

### Data extraction

Two reviewers conducted independently the data extraction using a pre-designed form, which contained the name of the first author, study characteristics (publication date, nationality, study design, data-analysis, golden standard, and study duration), characteristics of participants (sample size, mean age, and intervention received), technical parameters (scanner modality, ligand dose, and image analysis), and diagnostic outcomes (TP, FP, FN, and TN). Disagreements were settled via discussion.

### Statistical analysis

All statistical analyses were conducted using Stata software (version 15.0, Stata Corporation, College Station, Texas, USA) and Meta-Disc (version 1.4). We measured the sensitivity (SEN) and specificity (SPE) based on the data extracted in a 2 × 2 table, and calculated the 95% confidence interval (95%CI) of each variable.. Calculation of SEN and SPE were according to the equation of true positive/(false positive + true negative) and true negative/(false negative + true positive) formulas [15]. Receiver operating characteristic (ROC) curve was provided, and area under the curve (AUC) was adopted for assessment of the diagnostic performance. When the AUROC is 100%, a diagnostic test is deemed finished. AUROC more than 90% was regarded as exceptional, and AUROC larger than 80% as good.

Heterogeneity test was conducted using *I*^2^ statistic [16]. An *I*^2^ less than 50% with the *p* value greater than 0.1 indicated no significant heterogeneity considered among the included studies, then fixed-effect model would be adopted, otherwise (*I*^2^ greater than 50% with the *p* value less than 0.1), there would be significant heterogeneity and random-effect model would be used for meta-analysis [17, 18]. Spearman correlation coefficient was adopted for assessment of threshold-effect via Meta-Disc (version 1.4) [19]. Meta-regression and sensitivity analysis were performed for identifying and processing the heterogeneity [20]. A *p* value less than 0.05 would be considered statistically significant. Deeks' funnel plot was used for publication bias assessment. An asymmetrical would indicate significant publication bias. The degree of asymmetry was determined using DOR logarithm regression against half of the proper sample size. When looking at the slope coefficient, a* p* value less than 0.05 indicated a significantly asymmetric funnel plot [21].

## Results

### Literature search and study selection

**Fig. 1:**
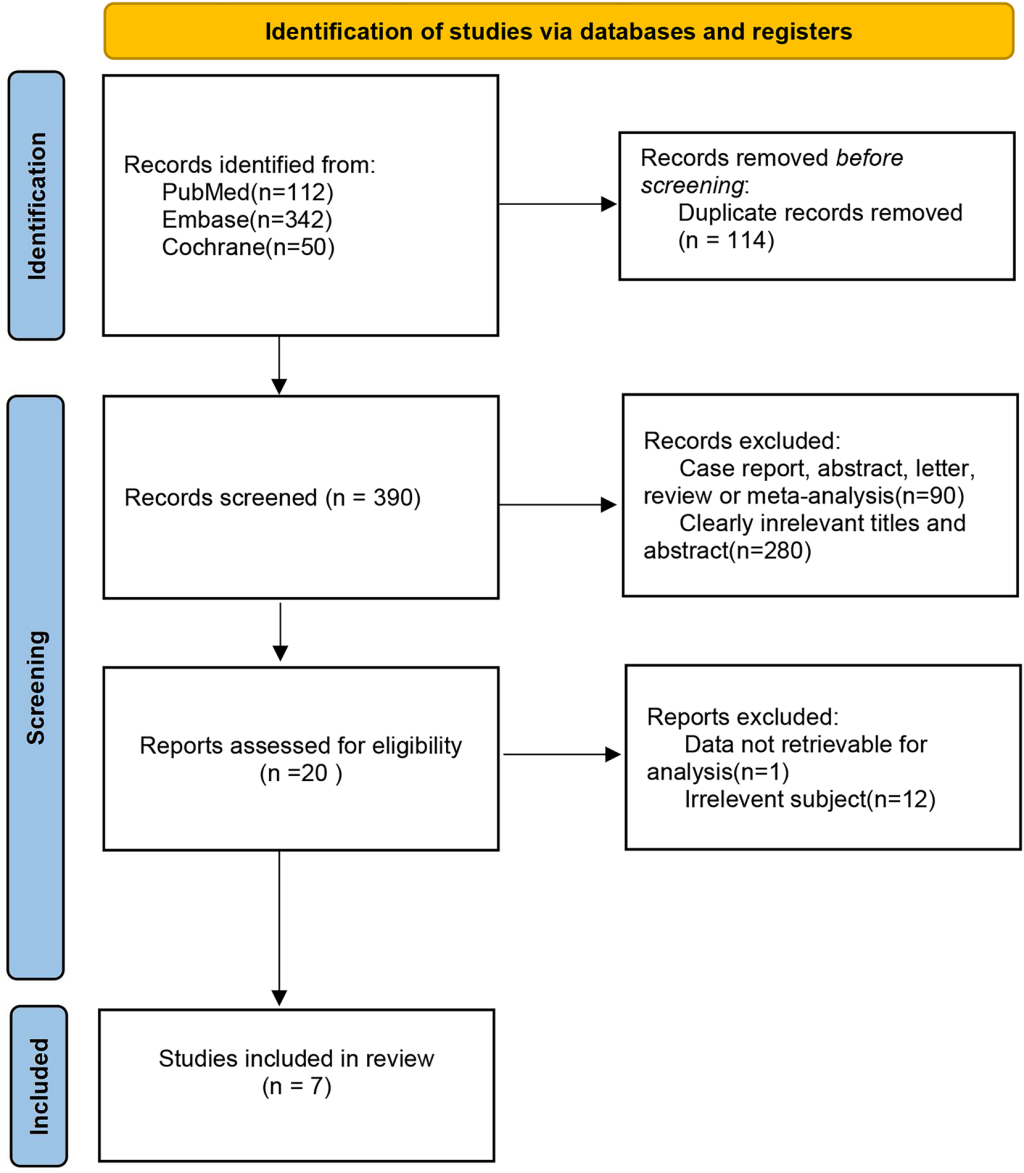
The PRISMA flow-diagram of study selection

There were totally 390 articles retrieved, and 370 were excluded. Full-texts of the rest 20 articles were read, and 13 studies were removed according to the following reasons: data unavailable for analysis (n = 1), irrelevant subjects (n = 12). Finally, 7 studies regarding the diagnostic efficacy of 18F-FDG PET/MRI for CRC LN-metastatic primary staging were included [13, 22–27]. The PRISMA flow-diagram of study selection is presented in Fig. 1.

### Characteristics and quality of included studies

**Fig. 2:**
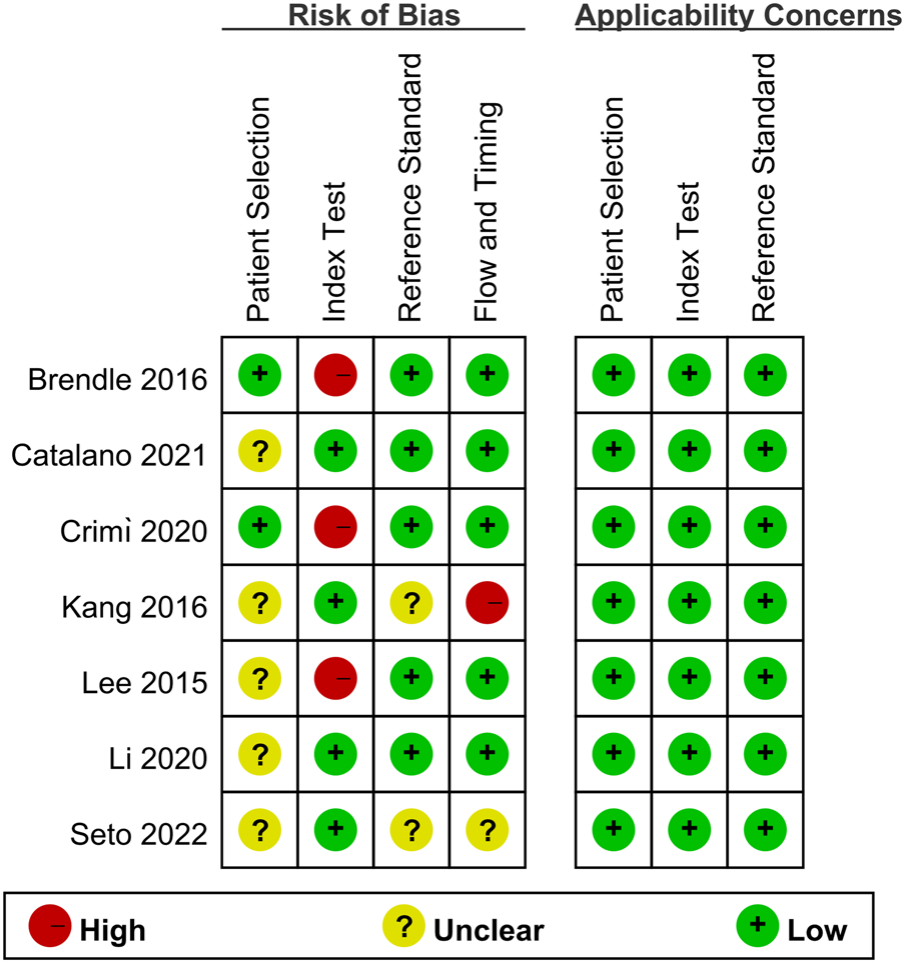
Summary risk of bias and applicability concerns of the included studies

Detailed characteristics of the studies are provided in Table 2. A total of 184 participants were involved. Table 3 provides the technical aspects of 18F-FDG PET/MRI. Results of risk of bias assessment are provided in Fig. 2, and all the 7 studies were graded as overall high quality.

**Table 2:** Study and patient characteristics of the included studies

Author	Year	Study characteristics					Patient characteristics		
		Country	Study design	Analysis	Gold standard	Study duration	No. of patients	Mean age ± SD	Treatment received (no. of pts)
Seto et al. [22]	2022	Japan	Retro	PB	Histopathology	2017–2019	16	NA	NA
Catalano et al. [23]	2021	American	Retro	PB	Histopathology Image follow-up	2016–2019	62	54 ± 12	Surg(29),NAG + Surg(14),Cx/Cx + RTx(19)
Crimì et al. [24]	2020	Italy	Pro	PB	Histopathology	2015–2018	36	68.5 (43–89)	Surg + Cx + RTx
Brendle et al. [25]	2016	Germany	Retro	LB	Image follow-up	NA	15	45.0(10–62)	Surg(1),Cx(3),Surg + Cx(9),Surg + Cx + RTx(1),untreated(1)
Kang et al. [13]	2016	Korea	Retro	PB	Histopathology	2012–2013	12	60.2 (34–81)	Surg(12)
Lee et al. [26]	2015	Korea	Pro	PB	Histopathology/image follow-up	2013	20	58.3 ± 12.0	Surg,NAG
Li et al. [27]	2020	Germany	Retro	PB	Histopathology	2012–2020	23	NA	NA

PB patient-based; LB lesion-based; Pro prospective; Retro retrospective; NAG neoadjuvant chemoradiation therapy; Cx chemotherapy; RTx radiotherapy

**Table 3:** Technical aspects of included studies

Author	Year	Scanner modality (PET/MRI)	Ligand dose	Image analysis	Total	TP	FP	FN	TN
Seto et al. [22]	2022	GE Healthcare,Signa PET/MR	200 MBq	Quantitative	16	8	0	1	7
Catalano et al. [23]	2021	Biograph mMR scanner Siemens Healthcare,Erlangen,Germany	4.44 MBq/kg	Quantitative	62	44	2	4	12
Crimì et al. [24]	2020	Biograph mMR scanner Siemens Healthcare,Erlangen,Germany	3 MBq/kg	Qualitative	36	10	2	1	23
Brendle et al. [25]	2016	Biograph mMR scanner Siemens Healthcare,Erlangen,Germany	NA	Qualitative	55	12	2	8	33
Kang et al. [13]	2016	Biograph mMR scanner Siemens Healthcare,Erlangen,Germany	5.18 MBq/kg	Quantitative	12	4	4	3	1
Lee et al. [26]	2015	Biograph mMR scanner Siemens Healthcare,Erlangen,Germany	5.18 MBq/kg	Qualitative	20	10	1	1	8
Li et al. [27]	2020	Biograph mMR scanner Siemens Healthcare,Erlangen,Germany	266.6 ± 58.8 MBq	Quantitative	23	7	1	4	11

### Diagnostic performance

**Fig. 3:**
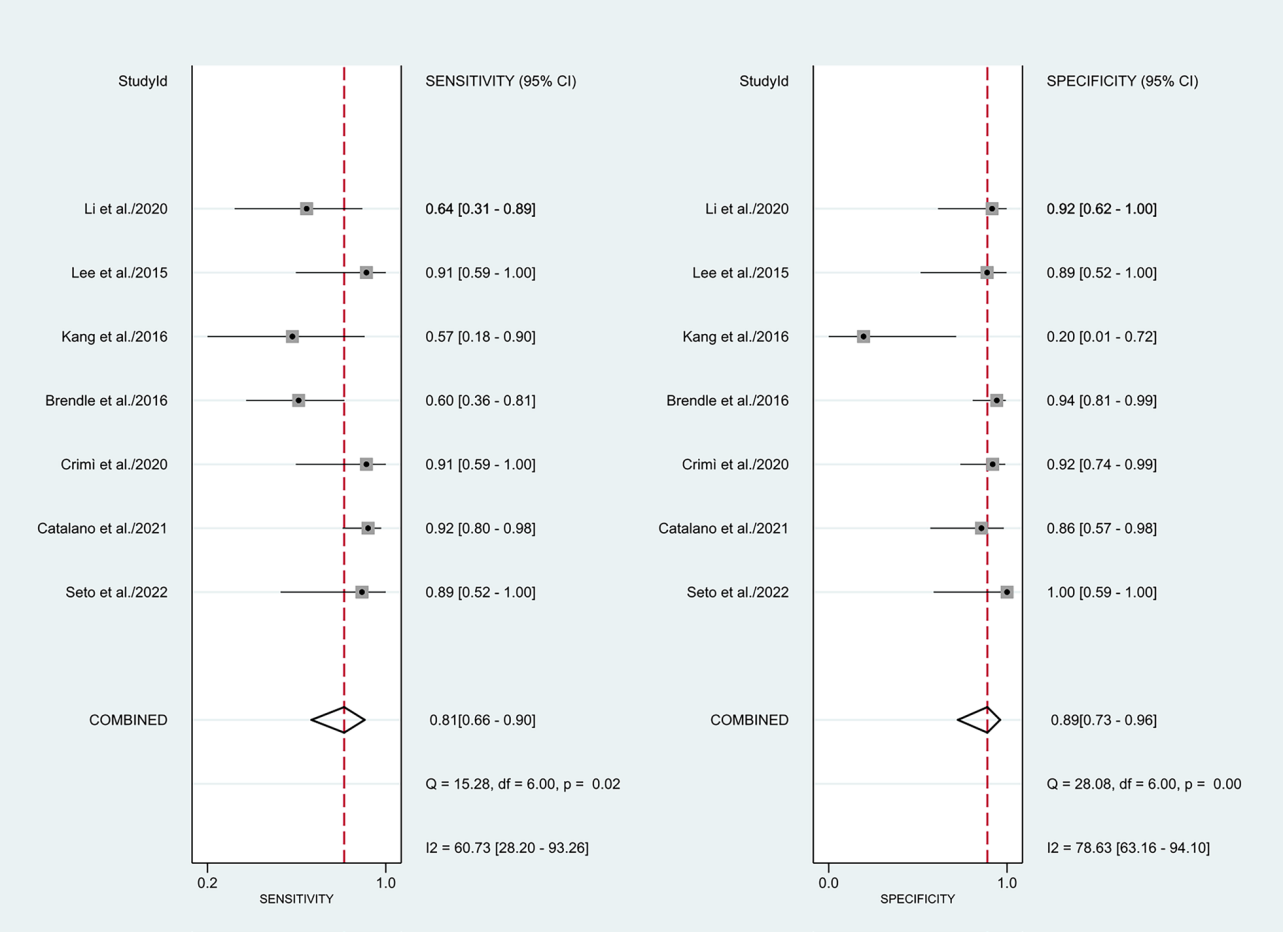
Forest plots for the pooled sensitivity and specificity calculation. Horizontal lines represent 95% CIs of the individual studies

**Fig. 4:**
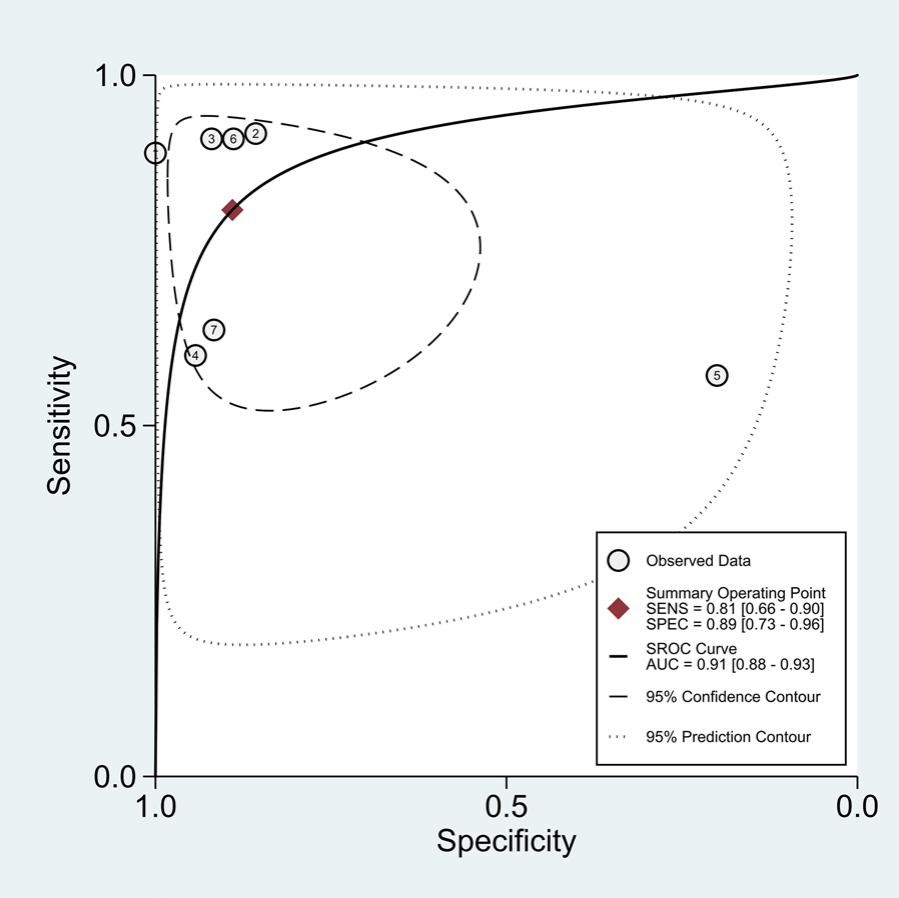
SROC curve of 18F-FDG PET/MRI for the detection of lymph node metastasis primary staging in colorectal cancer. The “Observed Data” points show accuracy for each study and the “Summary Operating Point” represents the pooled accuracy

**Fig. 5:**
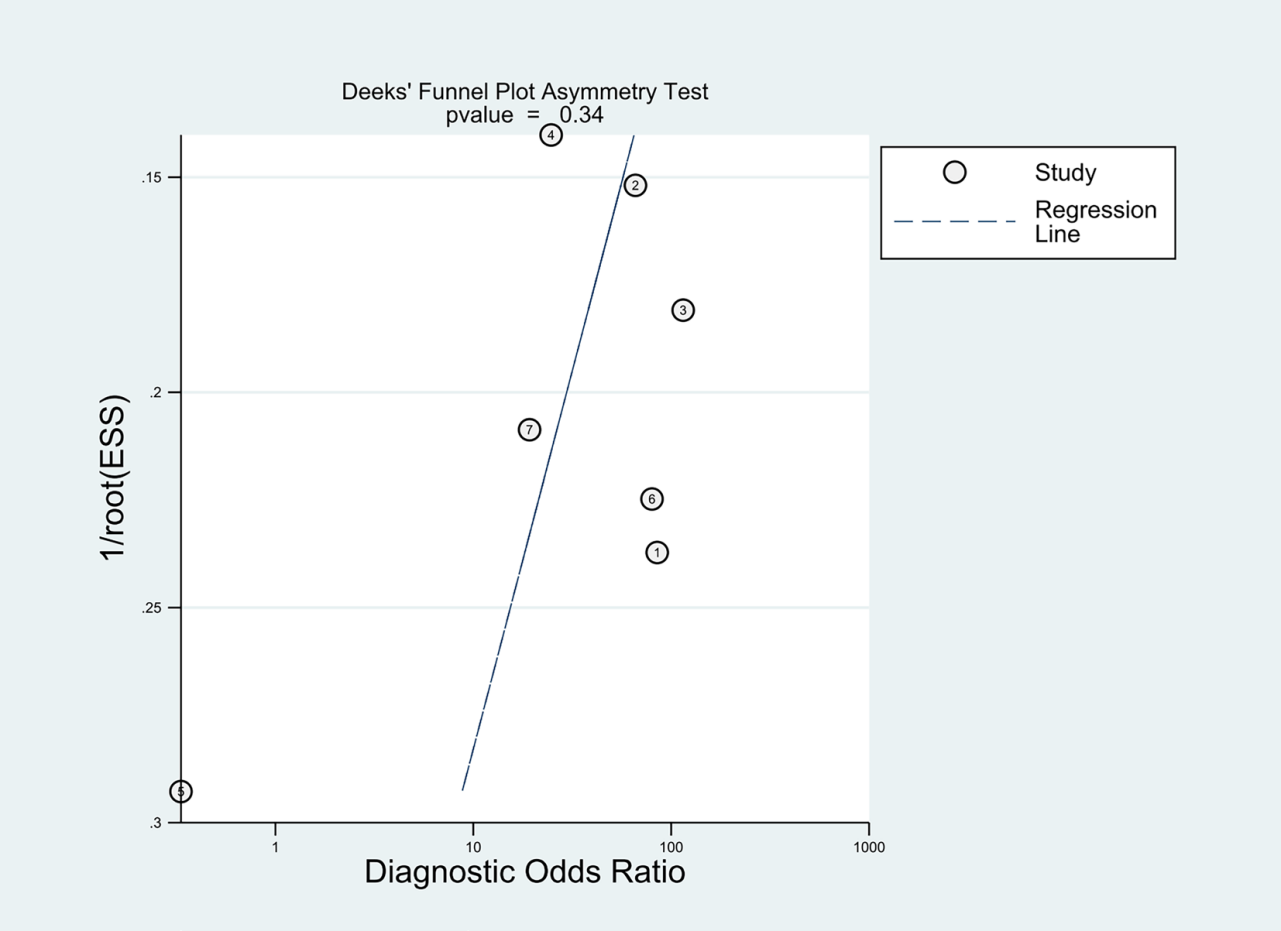
Effective sample size (ESS) funnel plot and the associated regression test of asymmetry. A *p* value < 0.10 was considered evidence of asymmetry and potential publication bias

**Fig. 6:**
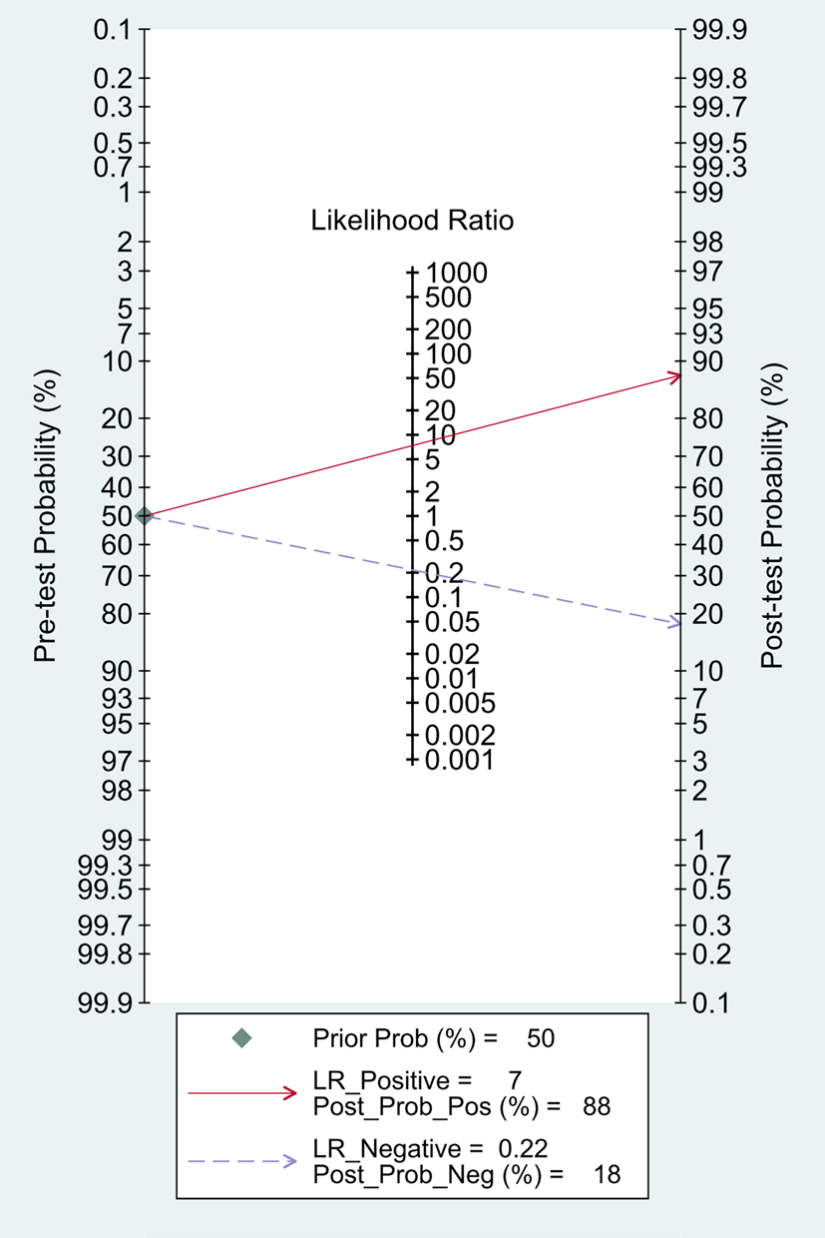
Fagan nomogram of pre-test probability, positive post-test probability and negative post-test probability for 18F-FDG PET/MRI. The pre-test probability was set at 50%

The Spearman rank correlation coefficient was 0.108 (*P* = 0.818), with no significant threshold-effect observed. The pooled SEN and SPE of 18F-FDG PET/MRI were 0.81 (95%CI 0.66–0.90) and 0.89 (95% CI 0.73–0.96), respectively, with intermediate heterogeneity (61% and 79%) (Fig. 3). The ROC curve is shown in Fig. 4, and the AUC of 18F-FDG PET/MRI was 0.91 (95% CI 0.88–0.93). No significant publication bias was identified according to the funnel plot (Fig. 5). Fagan's nomogram indicated that the post-test probability of 18F-FDG PET/MRI reached 88% as the pre-test probability set at 50% (Fig. 6).

### Heterogeneity analysis

Regarding the pooled SEN and SPE of 18F-FDG PET/MRI for the CRC LN-metastasis primary staging, the *I*^2^ was 60.73% and 78.63%, respectively. Subgroup analysis and meta-regression were conducted based on study characteristics, including sample size, race, and study design, to further identify the sources of heterogeneity, as shown in Table 4.

**Table 4:** Subgroup analysis of diagnostic performance of 18F-FDG PET/MRI

Covariate/Subgroup	Studies, *n*	Sensitivity (95% CI)	*P*-value	Specificity (95% CI)	*P*-value
Number of patients included			0.71		0.39
> 50	2	0.81 (0.61–1.00)		0.93 (0.80–1.00)	
≤ 50	5	0.81 (0.66–0.97)		0.87 (0.72–1.00)	
Ethnicity			0.96		0.12
Asian	3	0.83 (0.64–1.00)		0.80 (0.65–0.95)	
The rest	4	0.80 (0.65–0.95)		0.93 (0.85–1.00)	
Study design			0.22		0.38
Retro	5	0.76 (0.62–0.91)		0.87 (0.73–1.00)	
Pro	2	0.92 (0.79–1.00)		0.92 (0.78–1.00)	

The sources of heterogeneity were not identified after performing subgroup analysis and meta-regression, and sensitivity analysis was performed. Exclusion of the study by Catalano et al. from the 18F-FDG PET/MRI sensitivity analysis revealed a pooled SEN of 0.75 (95%CI 0.58–0.87), with acceptable heterogeneity (*I*^2^ = 39.52%). Removal of the study conducted by Kang et al. resulted in a pooled SPE of 0.92 (95%CI 0.84–0.96), with no significant heterogeneity (*I*^2^ = 0.00%). Finally, we determined that the studies conducted by Catalano et al. [23] and Kang et al. [13] would be the possible sources of heterogeneity for the diagnostic efficacy of 18F-FDG PET/ SEN MRIs and SPE, respectively.

## Discussion

We evaluated the diagnostic performance of 18F-FDG PET/MRI for the detection and staging of LN-metastasis in CRC. The overall pooled SEN and SPE were 0.81 and 0.89, respectively. It could provide accurate identification of malignancy in the lymph nodes that were under suspicion. Such an identification was unaffected by the treatment status of CRC patients.

The present study also provides a comprehensive overview of the application of 18F-FDG PET/MRI in LN-metastasis detection and CRC primary staging, and highlights aspects to which much attention should be paid. This would be significant for further studies and clinical practice. For instance, this study filled the gaps and addressed controversies that existed in previous studies, which might facilitate future exploration in this field. In addition, meta-analysis we conducted could provide, for the first time, significant information regarding the diagnostic accuracy of 18F-FDG PET/MRI in LN primary staging of CRC, which would be helpful for clinical decision-making and guide the use of this imaging modality in clinical practice. Informing diagnostic and treatment strategies of more remarkable effectiveness and efficiency could also improve the patients` outcomes.

Conventional treatments for CRC currently include radiotherapy and chemotherapy with 5-fluorouracil and mitomycin C. The dose of radiation for each LN is determined by its existence and size. The stage of LN-metastasis is one of the most critical indications in determining the necessity of adjuvant chemotherapy or LN dissection [3]. Accurate identification and assessment of CRC are prerequisite for effective treatment. Imageological examination can provide vital information for the disease location and distant involvement, as well as demarcation of the lesions [12]. CT is recommended as a standard imaging modality for initial cancer staging, follow-up reexamination, re-staging, and response-to-treatment evaluation in CRC patients due to its merits of being cost-saving and capability of full-body scanning [28]. MRI has the merits of widespread availability, excellent soft tissue contrast resolution, and validation in several trials [29]. Despite the widespread acceptance of CT and MRI for CRC regional staging and re-staging, their performance in LN metastasis identification is limited [30]. Meanwhile, there is conclusive evidence supporting a very high sensitivity of 0.939 for PET in CRC N-staging [31]. Combining CT with 18F-FDG PET (18F-FDG PET/CT) is used clinically for detecting nodal and distant metastases in high-risk CRC patients, but it is selected for regular CRC screening [30]. A meta-analysis regarding the use of PET/CT for clinical CRC N-staging showed an overall SEN of 42.9% and a SPE of 87.7%, which would be insufficient to support its routine use for CRC N-staging in clinical settings [32].

In contrast, integrated PET/MRI that combines MRI soft tissue morphology imaging with PET functional imaging is considered to be the most promising approach to address the problem mentioned above [33]. PET could provide extra information on the glycometabolism that is closely related to the potential of LN metastases, according to the findings of a pilot study on the colorectal staging and restaging following pCRT [34]. The introduction of a hybrid PET/MRI allows for the matching of the PET and MRI in one single examination, as well as comprehensive imaging that could help N-staging [12]. The use of PET/MRI is limited mainly by the lengthy acquisition and the high cost of examination, while patients could be free from other extra examinations after receiving PET/MRI so that the overall benefits would be considerable. Moreover, it is demonstrated that 18F-FDG PET/MRI is of more remarkable performance than 18F-FDG PET/CT or mono-MRI in specific clinical settings [12]. The results of this study have indicated that 18F-FDG PET/MRI has high sensitivity and specificity in detecting the presence of CRC, suggesting its potential to be used as an accurate imaging-detecting approach for CRC patients. Despite its expensive costs, patients receiving 18F-FDG PET/MRI could avoid multiple extra examinations and procedures. Our results indicated that the pooled SEN and SPE for CRC N-staging were 0.81 and 0.89, respectively. Adding metabolic components can improve the detective performance of MRI, especially the specificity.

Compared with the previous studies, our data indicated that the combination of 18F-FDG PET with MRI could significantly improve the diagnostic performance, and 18F-FDG PET/MRI would be more effective than other imaging modalities for nodal-staging. Heterogeneity was observed among the included studies, which might affect the pooled results. We conducted subgroup analysis and meta-regression based on the sample size, race, and study design. The sources of heterogeneity had not been identified. Therefore, the limited number of included studies and heterogeneity among the studies might limited the statistical power of the results. The study by Catalano et al. [23] reached a sensitivity of 92%, which was evidently different from the others and might cause heterogeneity due to its retrospective design and patients' inclusion bias. The study by Kang et al. [13] had a specificity of 20% of 18F-FDG PET/MRI in CRC primary LN-metastasis staging, which was considerably different from the others and might induce heterogeneity due to its retrospective design and limited sample size.

Several limitations exist in this study. Firstly, as previously stated, the significant heterogeneity among the studies necessitated caution when extrapolating the results in specific clinical settings. Although we have found that the study design and the limited number of patients would be associated with the heterogeneity, it might also be caused by other factors. Secondly, few studies were included in the meta-analysis, publication bias might be unavoidable. A per-patient meta-regression analysis is unfeasible due to limited studies included. Thirdly, more than half of the studies (4/7) were retrospective design so that the potential effects of PET imaging on CRC staging would be overestimated if the PET were conducted to scan suspected lesions under conventional imaging modalities, even though this was not indicated in any of the trials. Lastly, most of included studies took the imaging results produced during follow-up as the reference standard to avoid the possibility of unavailable histopathology, which compromises the performance of 18F-FDG PET/MRI, and subgroup analysis for pathological information is also unfeasible.

To sum up, this meta-analysis has proved that 18F-FDG PET/MRI has remarkable diagnostic performance in identification of LN metastases in newly diagnosed CRC patients. It is of great application value for primary staging of LN metastases in CRC.

## Data Availability

The data used in this study are available on request from the corresponding author.
